# Prognostic Significance of Glypican-3 Expression in Hepatocellular Carcinoma Treated with Atezolizumab-Bevacizumab

**DOI:** 10.3390/cancers17243967

**Published:** 2025-12-12

**Authors:** Ji Hoon Kim, Ji Won Han, Hee Sun Cho, Jeong Won Jang, Kwon Yong Tak, Pil Soo Sung

**Affiliations:** 1 The Liver Research Center, The Catholic University of Korea, Seoul 06591, Republic of Korea; jihoon23@gmail.com (J.H.K.); tmznjf@catholic.ac.kr (J.W.H.);; 2 Department of Gastroenterology and Hepatology, Seoul St Mary’s Hospital, The Catholic University of Korea, Seoul 06591, Republic of Korea

**Keywords:** GPC3, hepatocellular carcinoma, atezolizumab–bevacizumab, prognosis, biomarkers

## Abstract

**Background**: Glypican-3 (GPC3) is overexpressed in most hepatocellular carcinoma (HCC) tissues but is absent in normal adult liver. We evaluated whether tumor GPC3 expression is associated with clinical outcomes in patients with advanced HCC treated with atezolizumab–bevacizumab (AB). **Methods**: We conducted a single-center retrospective cohort study of 139 patients with Barcelona Clinic Liver Cancer (BCLC) stage C HCC who received AB between January 2022 and August 2025. Tumor GPC3 expression was assessed by immunohistochemistry. The primary endpoints were overall survival (OS) and progression-free survival (PFS), and the secondary endpoint was objective response rate (ORR) according to modified Response Evaluation Criteria in Solid Tumors (mRECIST). **Results**: Baseline characteristics were largely balanced between GPC3-positive (n = 87) and GPC3-negative (n = 52) groups. Median OS was significantly shorter in patients with GPC3-positive tumors than in those with GPC3-negative tumors (*p* = 0.006). In multivariable analysis, GPC3 positivity remained independently associated with higher mortality (hazard ratio [HR] 1.77, 95% confidence interval [CI] 1.05–3.00; *p* = 0.033), along with Child–Pugh class B. PFS did not differ significantly between the groups (*p* = 0.712). ORR was lower in GPC3-positive tumors than in GPC3-negative tumors (approximately 17–18% vs. ~32%; *p* = 0.023). Membranous GPC3 localization was associated with inferior OS compared with cytoplasmic or absent expression (*p* = 0.025). **Conclusions**: Tumor GPC3 expression was associated with decreased OS and lower ORR among AB-treated patients with advanced HCC, suggesting potential clinical relevance and may help in risk stratification.

## Simple Summary

Glypican 3 (GPC3) is highly expressed in most hepatocellular carcinomas (HCCs) but is absent in normal adult liver tissue. However, its clinical significance in patients treated with contemporary systemic therapies remains unclear. In this study, we evaluated 139 patients with advanced HCC who received atezolizumab plus bevacizumab as systemic therapy. We analyzed tumor biopsy samples to determine GPC3 expression patterns and investigated their association with clinical outcomes. Patients with GPC3-positive tumors showed significantly worse overall survival and a lower objective response rate compared with those with GPC3-negative tumors. Notably, membranous GPC3 expression was associated with the poorest survival outcomes. These findings indicate that tumor GPC3 expression is associated with poor clinical outcome with reduced benefit from immune–anti-angiogenic therapy. Our results highlight the potential role of GPC3 as a prognostic biomarker in advanced HCC.

## 1. Introduction

Hepatocellular carcinoma (HCC) is the most common primary malignancy of the liver and represents a major global health burden, with rising incidence and poor prognosis in advanced stages [1]. Although systemic therapies, including tyrosine kinase inhibitors and immune checkpoint inhibitors, have become the cornerstone of advanced HCC management, clinical outcomes remain highly heterogeneous, underscoring the need for robust prognostic and predictive biomarkers [2,3,4]. In fact, there have been incidences where even downstaging of HCC so transplantation could be done have been observed [5].

Glypican-3 (GPC3), an oncofetal heparan sulfate proteoglycan, is normally absent in the adult liver but is markedly upregulated in HCC. GPC3 contributes to oncogenesis via multiple signaling pathways, including Wnt/β-catenin and insulin-like growth factor (IGF) signaling [6]. Numerous studies have highlighted the diagnostic and therapeutic relevance of GPC3 expression in HCC. In 2022, Jiang et al. proposed GPC3 as a biomarker for HCC diagnosis and prognosis [7], and in 2020, Guo et al. showed that GPC3 is a promising candidate for HCC diagnosis and prediction of poor prognosis [8]. High GPC3 expression is detected in more than 70% of HCC tumors, making it one of the most consistently upregulated immunohistochemical markers in this disease [6,9]. Importantly, GPC3 overexpression has been associated with worse overall and disease-free survival, suggesting its potential role as a negative prognostic biomarker; however, its predictive value in the era of modern systemic therapies remains to be fully defined [9]. The significance of GPC3 expression in HCC is further underscored by its role as a membrane antigen targeted by emerging therapies such as chimeric antigen receptor T (CAR T) cells [10,11,12].

Since 2020, the combination of atezolizumab and bevacizumab (AB), comprising an anti-PD-L1 antibody and an anti-vascular endothelial growth factor antibody, has emerged as a frontline standard systemic treatment for advanced HCC, improving survival outcomes compared with prior therapies. The IMbrave150 trial and subsequent real-world studies have demonstrated durable partial responses and long-term survival in subsets of patients treated with AB, highlighting the need for biomarkers that can predict treatment response and guide patient stratification to optimize therapy [13,14].

In this context, GPC3 is a promising candidate prognostic and predictive biomarker for patients receiving AB. Given its cancer-specific overexpression and oncogenic role, GPC3 expression may reflect more unfavorable clinical profile to immune checkpoint inhibition or anti-angiogenic therapy. Conversely, GPC3-positive tumors could be prioritized for emerging GPC3-targeted therapies, including monoclonal antibodies and CAR T-cell approaches, providing a rationale for personalized treatment strategies [6]. However, in patients treated with modern immune-based systemic therapies, the clinical utility of GPC3 immunohistochemistry—specifically for prognostication or therapeutic prediction—remains incompletely characterized.

To address these gaps, we performed a retrospective cohort study to assess the prognostic and predictive significance of tumor GPC3 expression in patients with advanced HCC treated with AB combination therapy. This study aimed to inform risk stratification, guide clinical decision-making, and support the integration of GPC3-targeted modalities in translational research and clinical practice, ultimately improving patient outcomes in the era of immune checkpoint blockade and anti-angiogenic therapy.

## 2. Materials and Methods

### 2.1. Study Design and Patients

We retrospectively analyzed 139 patients with advanced HCC (BCLC-C) who had available biopsy specimens for GPC3 immunohistochemistry and received AB therapy between January 2022 and August 2025. HCC was diagnosed according to international guidelines using contrast-enhanced computed tomography (CT) or magnetic resonance imaging (MRI). Eligible patients were required to have an Eastern Cooperative Oncology Group (ECOG) performance status of 0–1 and at least one post-treatment imaging evaluation. Patients with Child–Pugh class C liver function were excluded. All biopsy specimens used for GPC3 immunohistochemistry were obtained prior to treatment initiation, within one week before starting atezolizumab–bevacizumab.

### 2.2. Treatment and Response Evaluation

Patients received intravenous atezolizumab 1200 mg plus bevacizumab 15 mg/kg every 3 weeks, following the IMbrave150 regimen, until radiological progression or unacceptable toxicity. Radiologic assessments with dynamic CT or MRI using liver-specific contrast agents were performed approximately every three cycles (about every 9 weeks). Tumor response was evaluated according to mRECIST criteria. The median time to the first mRECIST evaluation was 71 days.

### 2.3. GPC3 Immunohistochemistry

Formalin-fixed, paraffin-embedded tumor tissues were stained with an anti-GPC3 antibody (clone 1G12, Cell Marque, Rocklin, CA, USA; dilution 1:50). Sections were deparaffinized (xylene; Sigma-Aldrich, St. Louis, MO, USA), treated with 3% hydrogen peroxide (Daejung Chemicals, Siheung, Republic of Korea), and subjected to citrate buffer-based antigen retrieval (pH 6.0, Dako, Glostrup, Denmark). Slides were incubated overnight at 4 °C with the primary antibody, followed by Envision Plus HRP system (EnVision+ System-HRP, Dako, Glostrup, Denmark) detection and visualization with diaminobenzidine (DAB chromogen, Dako, Glostrup, Denmark). Hematoxylin (Sigma-Aldrich, St. Louis, MO, USA) was used as a counterstain. All slides were independently reviewed by pathologists blinded to the clinical data. GPC3 staining was assessed semiquantitatively by the pathologists using light microscopy. Staining intensity was graded on a standard semi-quantitative scale: 0 = no staining, 1+ = weak, 2+ = moderate, and 3+ = strong cytoplasmic or membranous staining. The H-score was calculated by multiplying the intensity (0–3) by the percentage of positive cells (0–100), yielding a score ranging from 0 to 300. GPC3 expression was considered positive when the H-score was > 0; cases with an H-score of 0 were classified as GPC3-negative. For subcellular localization analyses, tumors were further categorized into three patterns according to the predominant staining distribution. Membranous GPC3 expression was defined as distinct circumferential or basolateral membranous staining in ≥10% of tumor cells, with or without cytoplasmic staining. Cytoplasmic GPC3 expression was defined as granular or diffuse cytoplasmic staining in ≥10% of tumor cells without a clear membranous accentuation. Tumors without appreciable membranous or cytoplasmic staining in tumor cells were classified as GPC3-negative. Any discrepant interpretations between observers were resolved by joint review to reach a consensus.

### 2.4. Endpoints

The primary endpoints were overall survival (OS), defined as the time from initiation of AB treatment to death from any cause, and progression-free survival (PFS), defined as the time from treatment initiation to progressive disease according to mRECIST or death. Patients without events were censored at the last follow-up. The secondary endpoint was the objective response rate (ORR), defined as the proportion of patients achieving complete or partial response according to mRECIST.

### 2.5. Statistical Analysis

Baseline characteristics, including age, sex, Child–Pugh class, tumor stage, and prior therapies, were collected from medical records. Group comparisons according to GPC3 expression were performed using the chi-square or Fisher’s exact test for categorical variables and Student’s *t*-test or the Mann–Whitney U-test for continuous variables, as appropriate. OS and PFS were estimated using the Kaplan–Meier method and compared using the log-rank test. ORR was calculated among patients with evaluable imaging.

Multivariable Cox proportional hazards regression models were used to identify independent predictors of OS and PFS after adjusting for clinically relevant baseline factors. Results are reported as hazard ratios (HRs) with 95% confidence intervals (CIs). A two-sided *p*-value < 0.05 was considered statistically significant. All analysis was done using GraphPad Prism (GraphPad Software, San Diego, CA, USA).

## 3. Results

### 3.1. Baseline Characteristics According to Tumor GPC3 Expression

A total of 139 patients met the inclusion criteria and were included in the analysis. Baseline characteristics are summarized in Table 1. The mean age was 63.4 years, and most patients were male (83.5%). The predominant etiology of HCC was chronic hepatitis B virus infection (62.6%). Regarding liver function, 89.9% and 10.1% of patients were classified as Child–Pugh class A and B, respectively. All patients had Barcelona Clinic Liver Cancer (BCLC) stage C and 60.4% presented with portal vein tumor thrombosis (PVTT). The median AFP level was 479.0 ng/mL (IQR 17.7–14,211.5) and the median PIVKA-II level was 2639.0 mAU/mL (IQR 228.5–15,858.0). ECOG performance status was 0 in 67.6%, 1 in 30.9%, and 2 in 1.4% of patients. AST levels above 250 U/L were observed in 4.3% of the cohort and GGT above 800 U/L in 2.2%. Regarding treatment line, 51.1% of patients received atezolizumab–bevacizumab as first-line therapy. Second-line patients had received prior locoregional treatments (e.g., TACE) but did not receive any prior systemic therapy; therefore, all patients in this study received atezolizumab–bevacizumab as their first systemic treatment.

**Figure 1:**
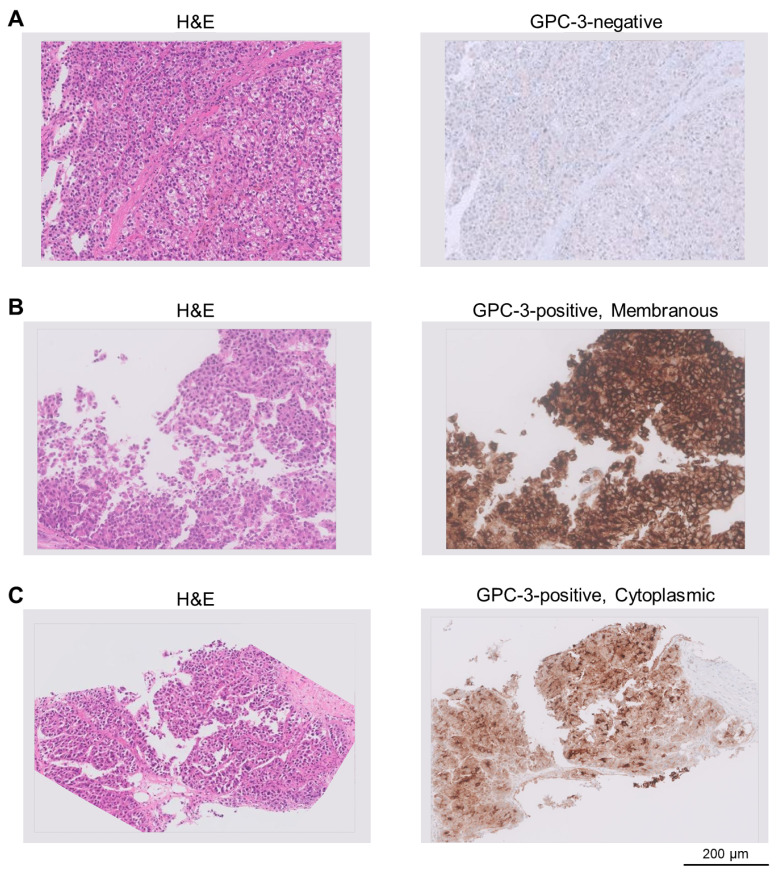
Representative histologic and immunohistochemical findings of hepatocellular carcinoma (HCC). Hematoxylin and eosin (H&E) staining (left) and glypican-3 (GPC3) immunohistochemistry (right). (**A**) GPC3-negative HCC tissue. (**B**) GPC3-positive HCC with a predominant membranous staining pattern. (**C**) GPC3-positive HCC with a cytoplasmic staining pattern.

Patients were categorized according to GPC3 expression in tumor tissue (Figure 1). Baseline characteristics, including age, sex, underlying liver function, and tumor stage, were well balanced between the GPC3-positive and GPC3-negative cohorts, minimizing confounding due to baseline imbalances. However, AFP levels were significantly higher in the GPC3-positive group, consistent with the association between GPC3 expression and elevated tumor marker burden.

### 3.2. Tumor GPC3 Expression Has Negative Impact on Patient Survival

**Figure 2:**
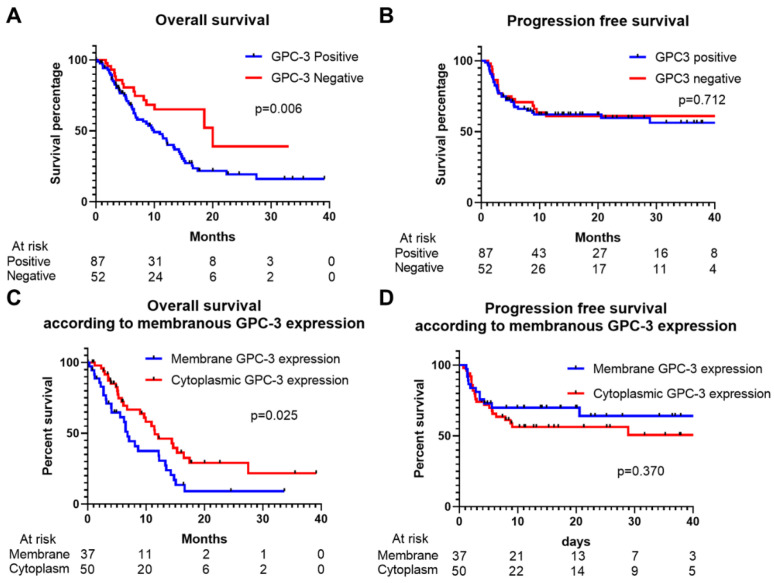
Kaplan–Meier survival analysis according to glypican-3 (GPC3) expression in hepatocellular carcinoma (HCC). (**A**) Overall survival comparison between patients with and without GPC3 expression (*p* = 0.006). (**B**) Progression-free survival comparison between patients with and without GPC3 expression (*p* = 0.712). (**C**) Overall survival comparison according to membranous versus cytoplasmic GPC3 expression (*p* = 0.025). (**D**) Progression-free survival comparison according to membranous versus cytoplasmic GPC3 expression (*p* = 0.370).

Among the 139 patients with advanced HCC, GPC3 positivity was observed in a substantial subset and was significantly associated with adverse clinical outcomes. Survival analyses revealed that median OS was markedly shorter in patients with GPC3-positive tumors than in those with GPC3-negative tumors (10.4 vs. 20.3 months; log-rank *p* = 0.006), supporting the role of GPC3 as a negative prognostic biomarker (Figure 2). In multivariable Cox regression analysis, GPC3 positivity remained independently associated with increased mortality (HR 1.77, 95% CI 1.05–3.00; *p* = 0.033), along with Child–Pugh class B (HR 3.65, 95% CI 1.94–6.86; *p* < 0.001) and PIVKA-II (HR 1.87, 95% CI 1.09–3.21; *p* = 0.022) (Table 2).

In contrast, PFS did not differ significantly between GPC3-positive and GPC3-negative groups (5.0 vs. 5.7 months; *p* = 0.712), suggesting that GPC3 status may be more strongly associated with long-term survival than with short-term disease control.

### 3.3. Prognostic Impact of Membranous GPC3 Expression

The subcellular localization of GPC3 expression within tumor cells also had prognostic implications. Patients with membranous GPC3 expression (n = 37) showed significantly poorer OS compared with those with cytoplasmic GPC3 expression (n = 50), whereas no significant differences were observed in PFS (Figure 2). These findings indicate that membranous GPC3 expression may denote a biologically distinct and more aggressive tumor phenotype.

### 3.4. Association Between Tumor GPC3 Expression and Objective Response

**Figure 3:**
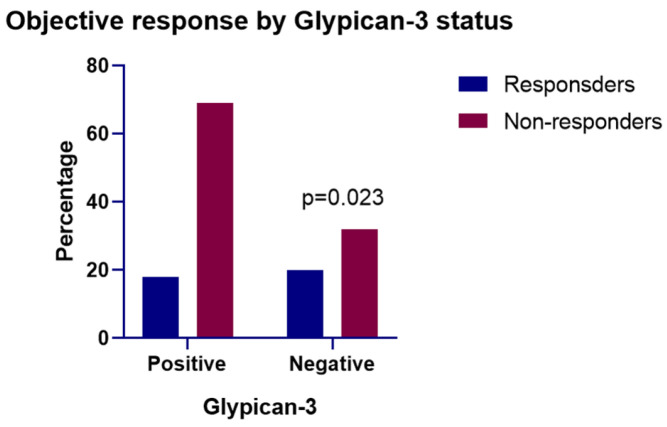
Objective response according to glypican-3 (GPC3) expression in hepatocellular carcinoma (HCC). Proportion of responders and non-responders stratified by tumor GPC3 expression status. GPC3-positive tumors showed a significantly lower objective response rate compared with GPC3-negative tumors (*p* = 0.023).

ORR analysis according to mRECIST revealed a lower response rate in patients with GPC3-positive tumors than in those with GPC3-negative tumors (20.7% vs. 38.5%; *p* = 0.023) (Table 3). This suggests that GPC3 expression is associated with a reduced likelihood of achieving radiologic tumor response to AB therapy (Figure 3). A chi-square test confirmed a statistically significant negative association between GPC3 positivity and treatment response.

## 4. Discussion

In this single-center cohort study of patients with unresectable HCC treated with AB combination therapy, we demonstrated that tumor GPC3 expression was significantly associated with worse OS and lower ORR, but not with PFS. Notably, GPC3 positivity was linked to reduced response rates despite similar PFS between groups. One potential explanation is that disease control rates may have been comparable, whereas GPC3-positive tumors achieved weaker depth or durability of response to systemic therapy, ultimately affecting long-term survival without altering median PFS. Additionally, this discrepancy may reflect the limited sensitivity of conventional radiographic criteria in capturing underlying tumor biology, particularly in patients receiving immunotherapy-based regimens. These observations underscore the need for biomarkers beyond traditional radiographic endpoints to better stratify treatment outcomes in HCC.

Our subgroup analysis revealed that membranous GPC3 expression was associated with poorer OS compared with cytoplasmic or absent expression, suggesting that subcellular localization carries additional prognostic information. Given that GPC3 is a membrane-bound proteoglycan and a target for emerging immunotherapies, membranous expression may mark a more aggressive tumor phenotype that is less responsive to immune checkpoint and anti-angiogenic therapies. Previous pathological studies have suggested that subcellular GPC3 localization may have distinct biological implications: membranous GPC3 expression has been linked to poor differentiation and microvascular invasion, whereas cytoplasmic expression has been linked to well-differentiated tumor histology and may reflect a more immune-accessible phenotype. Kaseb et al. reported that GPC3 expression correlated with a higher risk of recurrence and worse OS, and part of their data suggests that membranous GPC3 expression is associated with poorer outcomes than cytoplasmic or mixed GPC3 expression [15]. Together with our results, these patterns support the notion that membranous GPC3 may be biologically less favorable and potentially less susceptible to immune checkpoint-based regimens such as AB, highlighting the importance of evaluating GPC3 localization in addition to overall positivity.

To our knowledge, this is among the first real-world studies to provide evidence that immunohistochemically detected GPC3 expression is related to clinical outcome for systemic treatment of advanced HCC. We found that GPC3 positivity was independently associated with decreased OS and lower ORR in patients treated with AB, supporting its clinical utility for risk stratification in advanced HCC. These findings are consistent with prior cohorts and meta-analyses in which GPC3 overexpression was associated with aggressive tumor biology and poor long-term outcomes [7,9]. The observed association between GPC3 expression and inferior survival aligns with preclinical and translational data implicating GPC3 in tumor growth, angiogenesis, and activation of oncogenic pathways such as WnWnt-catenin and IGF signaling [7,9]. In 2016, Kaseb et al. showed that higher GPC3 expression was associated with worse prognosis across BCLC stages [11], and in 2014, Xiao et al. demonstrated that GPC3 overexpression correlated with poor prognosis in HCC [16]. High GPC3 expression has also been linked to vascular invasion and early recurrence in resectable HCC. Feng et al. reported that GPC3 and cytokeratin-19 can serve as biomarkers for predicting poor prognosis and early recurrence after radical resection [17], and Hamaoka et al. demonstrated that preoperative GPC3 positivity was a significant risk factor for PVTT and poor prognosis [15]. Our findings extend this body of evidence to a population treated with modern systemic immunotherapy, supporting GPC3 as both a diagnostic marker and a biomarker of poor clinical outcomes in unresectable disease.

The identification of GPC3 as a negative biomarker has potential clinical implications. Patients with GPC3-positive tumors may represent a high-risk subgroup requiring intensified surveillance, earlier clinical trial enrollment, or rational combination strategies. Furthermore, GPC3 has emerged as a therapeutic target, with ongoing trials of GPC3-directed therapies including monoclonal antibodies, antibody–drug conjugates, CAR T-cell therapies, and peptide vaccines [6,10,18]. Our results reinforce the biological and clinical rationale for these approaches and support a role for GPC3 as a prognostic marker in advanced HCC. Beyond GPC3, additional molecular markers have been proposed as clinically relevant in HCC. Traditional serum biomarkers, such as alpha-fetoprotein (AFP) and des-gamma-carboxy prothrombin, and imaging features remain useful but lack specificity and consistency for prognostic prediction [19,20,21,22]. Recently, novel protein and transcriptomic candidates such as CKAP4 and PLOD2 have been identified as being upregulated in HCC tissue, with their combined overexpression acting as an independent predictor of worse prognosis [23,24,25,26,27]. Multigene signatures, including BR*D4*-interacting genes (e.g., *EZH2*, *KIF20A*, *G6PD*, and *KIF2C*), further highlight the biological complexity and heterogeneity of HCC and reinforce the need for integrated molecular approaches to complement clinicopathological staging [28,29,30].

Immune markers are of particular interest in the era of immune checkpoint inhibitors for HCC [31,32]. Although PD-L1 expression has clear prognostic utility in several malignancies, its significance in HCC—particularly in patients treated with AB—remains uncertain due to limited and sometimes conflicting evidence [33,34,35]. Some studies have suggested that elevated tumoral PD-L1 levels may be associated with poorer prognosis; however, large clinical trials, including IMbrave150, did not require PD-L1 selection and did not identify a clear predictive value for PD-L1 in selecting responders [36]. Other immune-related factors, such as tumor-infiltrating lymphocytes, immune gene signatures, and markers of neoangiogenesis, are promising but require further validation before routine clinical implementation [37,38,39,40,41]. In this context, GPC3 may provide complementary information, reflecting both tumor biology and potential eligibility for GPC3-directed therapies.

In interpreting our findings, several limitations should be noted. The modest sample size may limit the maturity and stability of time-to-event estimates, and the lack of a PFS difference suggests that imaging-based progression alone may not fully capture the biological differences associated with GPC3 expression. The high absolute AFP and PIVKA-II levels and their discrepancies between patient subgroups also warrant cautious interpretation. Finally, the retrospective, single-center nature of this study limits generalizability, and prospective validation in larger, multicenter cohorts—along with mechanistic investigation into the biological role of GPC3 in treatment response—is needed.

## 5. Conclusions

In conclusion, our study identifies GPC3 expression as an independent adverse prognostic factor and associated with differences in response in patients with advanced HCC treated with AB. These findings underscore the need for further prospective investigations and may inform patient stratification as well as the development and integration of GPC3-targeted therapies. Future research should prioritize large validation cohorts and explore combinatorial biomarker approaches that integrate GPC3 with other molecular and immune markers to refine decision-making and improve outcomes in advanced HCC.

## Figures and Tables

**Table 1:** Baseline characteristics of the overall patient population.

Variable	Total (n = 139)	GPC3 Positive (n = 87)	GPC3 Negative (n = 52)	*p*-Value
Age	63.4 ± 11.5	62.7 ± 12.2	64.6 ± 10.4	0.334
Sex				0.961
Male	116 (83.5)	72 (82.8)	44 (84.6)	
Female	23 (16.5)	15 (17.2)	8 (15.4)	
Etiology				0.553
HBV	87 (62.6)	53 (60.9)	34 (65.4)	
HCV	7 (5.0)	4 (4.6)	3 (5.8)	
Alcohol	27 (19.4)	20 (23.0)	7 (13.5)	
Other	18 (12.9)	10 (11.5)	10 (20.0)	
AFP (ng/mL)	479.0 (17.7–14,211.5)	1640.0 (45.3–34,137.5)	97.1 (6.45–1955.75)	0.003
PIVKA-II (mAU/mL)	2639.0 (228.5–15,858.0)	3245.0 (351.0–25,968.5)	1408.0 (76.5–9927.5)	0.040
AST (U/L)				0.132
>250	6 (4.3)	6 (6.9)	0 (0)	
≤250	133 (95.7)	81 (93.1)	52 (100)	
GGT ^†^ (U/L)				0.293
>800	3 (2.2)	3 (3.4)	0 (0)	
≤800	136 (97.8)	84 (96.6)	52 (100)	
ECOG				0.152
0	94 (67.6)	54 (62.1)	40 (76.9)	
1	43 (30.9)	32 (36.8)	11 (21.2)	
2	2 (1.4)	1 (1.1)	1 (1.9)	
Child–Pugh class				0.111
A	125 (89.9)	75 (86.2)	50 (96.2)	
B	14 (10.1)	12 (13.8)	2 (3.8)	
BCLC				1.000
C	139 (100)	87 (100)	52 (100)	
PVTT				0.740
No	55 (39.6)	33 (37.9)	22 (42.3)	
Yes	84 (60.4)	54 (62.1)	30 (57.7)	
Treatment line				0.742
First-line	71 (51.1)	43 (49.4)	28 (53.8)	
Second-line	68 (48.9)	44 (50.6)	24 (46.2)	

Data are presented as n (%), mean ± standard deviation, or median (interquartile range). ^†^ Data missing for 3 patients. AFP, alpha-fetoprotein; PIVKA-II, protein induced by vitamin K absence or antagonist-II; ECOG, Eastern Cooperative Oncology Group; BCLC, Barcelona Clinic Liver Cancer; PVTT, portal vein tumor thrombosis; HBV, hepatitis B virus; HCV, hepatitis C virus.

**Table 2:** Multivariate Cox regression analysis of overall survival.

	Univariate	Multivariate	
	*p*-value	HR (95% CI)	*p*-value
Glypican-3 (positive)	<0.001	1.77 (1.05–3.00)	0.033
Age	0.969		
Sex (Male)	0.623		
Child–Pugh (B)	<0.001	3.65 (1.94–6.86)	<0.001
AFP	<0.001	1.08 (0.63–1.84)	0.870
PIVKA-II	<0.001	1.87 (1.09–3.21)	0.022
PVTT	<0.001	1.09 (0.67–1.78)	0.723

An HR > 1 indicates an increased risk of death (worse overall survival). HR, hazard ratio; CI, confidence interval; BC P, alpha-fetoprotein; PVTT, portal vein tumor thrombosis.

**Table 3:** Tumor response evaluated with mRECIST.

	PR	SD/PD	Unknown
GPC3-positive (n = 87)	18 (20.7%)	69 (79.3%)	0
GPC3-negative (n = 52)	20 (38.5%)	23 (44.2%)	9 (17.3%)

ORR was calculated among patients with evaluable imaging; nine patients in the GPC3-negative group had unevaluable or missing response assessments and were excluded from analysis. GPC3, glypican-3; PR, partial response; SD, stable disease; PD, progressive disease; mRECIST, modified Response Evaluation Criteria for Solid Tumors.

## Data Availability

The datasets generated and analyzed in this study are not publicly available due to institutional and patient privacy regulations but are available from the corresponding authors upon reasonable request and subject to approval by the Institutional Review Board.

## Abbreviations

AB, atezolizumab–bevacizumab; AFP, alpha-fetoprotein; BCLC, Barcelona Clinic Liver Cancer; CI, confidence interval; CT, computed tomography; ECOG, Eastern Cooperative Oncology Group; GPC3, glypican-3; HBV, hepatitis B virus; HCC, hepatocellular carcinoma; HCV, hepatitis C virus; HR, hazard ratio; IGF, insulin-like growth factor; IHC, immunohistochemistry; MRI, magnetic resonance imaging; mRECIST, modified Response Evaluation Criteria in Solid Tumors; ORR, objective response rate; OS, overall survival; PD, progressive disease; PD-L1, programmed death-ligand 1; PFS, progression-free survival; PR, partial response; PVTT, portal vein tumor thrombosis; SD, stable disease; Wnt/β-catenin, Wingless/Integrated–β-catenin signaling pathway.
